# GtxA is a virulence factor that promotes a Th2-like response during *Gallibacterium anatis* infection in laying hens

**DOI:** 10.1186/s13567-020-00764-2

**Published:** 2020-03-11

**Authors:** Bo Tang, Susanne E. Pors, Bodil M. Kristensen, Ragnhild Bager J. Skjerning, Rikke H. Olsen, Anders M. Bojesen

**Affiliations:** Department of Veterinary and Animal Sciences, Faculty of Health and Medical Sciences, University of Copenhagen, Stigboejlen 4, 1870 Frederiksberg C, Denmark

## Abstract

GtxA, a leukotoxic RTX-toxin, has been proposed a main virulence factor of *Gallibacterium anatis*. To evaluate the impact of GtxA during infection, we experimentally infected laying hens with a *G. anatis* wild-type (WT) strain and its isogenic *gtxA* deletion mutant (Δ*gtxA*), respectively, and monitored the birds during a 6 day period. Birds inoculated with Δ*gtxA* had significantly reduced gross lesions and microscopic changes compared to the birds inoculated with the WT strain. To assess the host response further, we quantified the expression of pro-inflammatory cytokines and apoptosis genes by RT-qPCR. In the ovarian tissue, the expression levels of IL-4 and TNF-α were significantly lower in the Δ*gtxA* group compared to the WT group, while IL-6 and IL-10 levels appeared similar in the two groups. In the spleen tissue of Δ*gtxA* infected chickens, IL-4 expression was also lower compared to the WT infected chickens. The results indicated that GtxA plays a key role in an acute cytokine-mediated Th2-like response against *G. anatis* infection in the ovary tissue. The pro-inflammatory response in the ovary tissue of birds inoculated with Δ*gtxA* mutant was thus significantly lower than the wild-type response. This was, at least partly, supported by the apoptosis gene expression levels, which were significantly higher in the Δ*gtxA* mutant compared to the wild-type infected chickens. In conclusion, GtxA clearly plays an important role in the pathogenesis of *G. anatis* infections in laying hens. Further investigations into the specific factors regulating the host response is however needed to provide a more complete understanding of the bacteria-host interaction.

## Introduction

*Gallibacterium anatis* is a member of the *Pasteurellaceae* family colonizing the upper respiratory tract and lower genital tract of chickens [1]. Experimental infection studies have indicated that *G. anatis* has a pathogenic potential with a special affinity for the reproductive tract [2–4], and accumulating evidence points at *G. anatis* having an important role as a cause of salpingitis and peritonitis in laying hens worldwide, significantly compromising animal welfare and decreasing egg-yield [5–7].

Although some knowledge has been generated on specific factors important in the pathogenesis of *G. anatis* infections, several questions remain [8]. “Repeats in Toxins” (RTX-toxins) are potent cytolytic toxins produced by many Gram-negative bacteria including a wide range of species within the *Pasteurellaceae* family [9]. A *G. anatis* specific RTX-toxin (GtxA; *Gallibacterium* toxin) was identified by Kristensen et al. [10]. *Gallibacterium anatis* strains lacking parts of *gtxA* lost their haemolytic and cytolytic activity [11, 12]. Furthermore, GtxA has been shown to be immunogenic, and thus a promising vaccine candidate [13], which was partly confirmed by a study showing that the three recombinant proteins, GtxA-N, GtxA-C, and FlfA, could induce protection against *G. anatis* in an in vivo challenge model [14]. Several questions however remain regarding the role of GtxA in the pathogenesis of *G. anatis* [10].

The innate immune system utilizes pathogen-associated molecular patterns, such as Toll-like receptors (TLRs), which are expressed in chicken reproductive organs [15, 16] and play an essential role in the host defense mainly through immune recognition by the chicken [17]. Subsequently, a series of downstream cytokines, such as interferons (IFN) and interleukins (IL) are induced via cellular signaling pathways. Pathogenic *G. anatis* adhering to primary chicken oviduct epithelial cells have been shown to induce a strong inflammatory response and secretion of various cytokines, such as IL-6, TNF-α, and IFN-γ, which may lead to cell damage [18]. To what extend pathogenic *G. anatis* induces inflammatory cytokines in vivo has however not been reported.

Regulation of programmed cell death or apoptosis also plays an important role during bacteria-host interactions [19–21]. Many bacterial pathogens including *Escherichia coli* and *Salmonella* excrete pore-forming toxins that induce apoptosis, which may allow the pathogen to engage or circumvent the host’s efforts at limiting infections [22–24]. The importance of pore-forming toxins in *G. anatis*-induced apoptosis and their role in the pathogenesis has however not yet been clarified.

To investigate the role of GtxA during experimental infection in the natural host, we aimed at comparing lesions in laying hens infected with a *G. anatis* wildt-ype (WT) strain and its isogenic *gtxA* deletion mutant strain, which is unable to express GtxA. To assess the specific impact of GtxA on selected host immune factors, expression levels of genes encoding apoptosis and pro-inflammatory cytokines were assessed.

## Materials and methods

### Experimental animals and housing facilities

Twenty-four Lohmann Brown layer hens, 29-week old, were purchased from a commercial breeder with high biosecurity standards. At arrival, a swab from the cloacal mucosa was obtained from each chicken and immediately streaked on blood agar (BA) plate (Blood agar base, Difco, Heidelberg, Germany added 5% citrated bovine blood) The plates were incubated in a sealed plastic bag at 37 °C for 18 h. All plates were examined and presumptive *G. anatis* colonies were confirmed by PCR [25]. One colony of confirmed *G. anatis* growth from each bird was picked and prepared for storage at −80 °C for later use. The hens were kept under free indoor housing conditions with controlled ventilation, humidity and temperature, and were provided with fresh water and feed ad libitum. The hens were allowed to acclimatize for 1 week prior to the trial. All work with the chickens was carried out with the approval of the Danish Animal Experiments Inspectorate (license number: 2012-15-2934-00339).

### Bacterial strains and experimental infection

A GtxA deletion mutant, *G. anatis* Δ*gtxA*, constructed as previously reported by Kristensen et al. [10] and its virulent parent wild-type (WT) strain *G. anatis* (strain no. 12656-12) were used for the inoculations. The WT strain was originally isolated from a bird with septicaemia. The strain has been characterized in detail by phenotypic and genotypic methods and has previously been used for in vivo studies of pathogenicity [2, 26]. Both strains were stored at −80 °C and cultivated overnight on BA in a sealed plastic bag to obtain single colonies, which were subsequently incubated in Brain Heart Infusion (BHI) broth (Oxoid, Basingstoke, UK) with agitation at 37 °C overnight. Prior to inoculation, an overnight culture of each strain was added to fresh BHI (in dilution ratio 1:2) followed by incubation at 37 °C for approximately three hours to reach the late log-phase and a concentration of approximately 10^9^ CFU/mL. The bacterial concentration in each inoculum was verified by plate counts of tenfold serial dilutions of inocula on BA plates in duplicate. For the infection study, birds were randomly assigned to one of three groups (Table 1). The *G. anatis* Δ*gtxA* infection group was inoculated with a single dose of 4.9 × 10^8^ CFU (in 1 mL BHI). The *G. anatis* WT infection group received a single dose of 5 × 10^8^ CFU (in 1 mL BHI). The inocula were injected into the peritoneal cavity, just ventral for the spine and caudal to the last rib, using a 25 G cannula with a 30 mm-length. Chickens in the control group served as uninfected controls and were inoculated with 1 mL of sterile BHI. The unequal distribution of chickens in the *gtxA* mutant and wild-type groups (16 vs 4, respectively) was based on several previous studies done with the *G. anatis* WT (strain 12656-12), which made infections with this strain highly predictable and thus allowed fewer birds to be included [2, 14, 27].

**Table 1:** **Design of the study. A total of 24 birds were inoculated with*G. anatis*Δ*gtxA*(16 chickens),*G. anatis*12656-12 (4 chickens) or BHI (4 chickens)**

Group	Inoculum	Dose/CFU	Chickens examined post-inoculation (pi)	
			2 days pi	6 days pi
*G. anatis* Δ*gtxA*	Δ*gtxA*	4.9 × 10^8^	8	8
*G. anatis* wild-type	12656-12	5.0 × 10^8^	2	2
Control	BHI	–	2	2

### Post-mortem examination

The chickens were euthanized and subjected to post-mortem examination at either 2 or 6 days post-infection (pi) (Table 1). Recording of gross lesions and tissue sampling for histology was done for selected organs including the spleen, liver, ovary and oviduct. Tissue samples from the spleen and ovary were stored in RNAlater (Qiagen, Hilden, Germany) according to the manufacturer’s instructions. Bacterial isolates from the spleen, liver, ovary and salpinx were obtained by streaking tissue swabs onto BA. Isolates of the *G. anatis* WT strain were identified as *G. anatis* when greyish, semi-transparent colonies surrounded by a haemolytic zone of 1 to 2 mm and had phenotypic characters resembling those previously reported [28]. *Gallibacterium anatis* Δ*gtxA* showed identical characteristics but lacked a haemolytic zone [10]. Tissue samples from the ovary and oviduct were fixed in 10% neutral buffered formalin for 24 h, processed by standard histological procedures through graded concentrations of ethanol and xylene, and finally embedded in paraffin wax, sectioned 3–5 µm thick, stained with haematoxylin and eosin, and evaluated by light microscopy (Olympus BX45). Samples from all birds were examined for histological lesions together with the controls and the inflammatory reactions, including cellular infiltration, edema, exudation and necrosis, were evaluated according to extent and distribution. The lesions were differentiated using semi-quantitative grading system where scored 0 (no lesions), + (few scattered lesions), ++ (moderate number lesions), and +++ (severe diffuse lesions).

### Pulsed-field gel electrophoresis (PFGE)

To verify that the re-isolated *G. anatis* from lesions were identical to the inoculum and different from the resident cloacal strains, pairs of *G. anatis* obtained from the cloaca and from lesions from eleven birds were characterized by pulsed-field gel electrophoresis (PFGE). The isolates had been kept at −80 °C in BHI broth with 10% glycerol and were plated on BA. Inoculated plates were incubated at 37 °C in sealed plastic bags for 18 h. From pure cultures, a single colony typical of *G. anatis* was picked and incubated overnight in 10 mL BHI broth at 37 °C with shaking. The preparation of bacterial DNA and separation of fragments was done as described [30] and using *Sal*I (R0114, New England BioLabs) and *Xba*I (R0145, New England BioLabs) as restriction enzymes.

### Serum sampling and enzyme-linked immunosorbent assay (ELISA)

GtxA specific antibody levels were quantified in serum samples by ELISA. Blood, 1–2 mL, was taken from *vena brachialis* from all birds at the day of inoculation (Day 0) and at 2 and 6 days after inoculation, respectively, and left at 4 °C overnight. Serum was collected after centrifugation at 1800 *g* for 10 min and stored at −20 °C. Two microtiter plate wells (Nunc-Immuno™ MicroWell™ 96-Well Plates, Thermo Scientific) were coated overnight at 4 °C with 0.5 µg GtxA recombinant protein diluted in carbonate-bicarbonate buffer (pH 9.6) (Sigma-Aldrich). Each well was then washed; this and all subsequent washing steps consisted of three washes in 350 µL wash buffer (PBS + 0.05% Tween 20) [13]. The wells were blocked for 2 h at room temperature in 200 µL blocking solution (PBS containing 0.05% Tween 20 and 2% bovine serum albumin (BSA)) and washed. The antibody titers were assayed by serial threefold dilutions of chicken serum ranging from 1:200 to 1:48 600. All dilutions were prepared in triplicates in a dilution buffer (PBS containing 0.05% Tween 20 and 0.1% BSA), 100 µL was added to each well and plates were incubated for 1 h at 37 °C. For each assay, 12 control wells were included, which contained pure dilution buffer; secondary antibody was added to 6 of these wells as a measure of background, and the other 6 wells remained blank as a negative control for the ELISA. Following incubation, the wells were washed and 100 µL polyclonal goat anti-chicken IgG (Fc): HRP (AbD Serotec) diluted 1:4000 in diluting buffer were added to each well and the plates incubated for a 1 h at 37 °C and then washed. To detect binding, 100 µL of 3,3′,5,5′-Tetramethylbenzidine liquid substrate (Sigma) were added to each well. The plates were incubated for 2 min and then the reaction was stopped by addition of 100 µL 1 M HCl. The absorbance was read immediately at 450 in a PowerWave XS spectrophotometry (BioTek Instruments).

### Differential gene expression analysis by Real-Time Quantitative PCR (RT-qPCR)

To examine the mRNA expression profiles in the spleen and ovary tissue samples obtained during necropsy and placed in RNA later and stored according to the manufacturer’s instructions (Merck Life Science A/S, Søborg, Denmark). For RNA extraction a total of 20 mg tissue from each organ was placed in 1.5 mL tube containing beads. Each sample was then homogenized in 1 mL lysate buffer RLT using a Vortex adapter (Mo Bio, Carlsbad, CA, USA) and centrifugated at 1800 *g*. After homogenization and centrifugation for 5 min, the upper phase of total RNA was collected and purified with an RNeasy mini kit (Qiagen, Hilden, Germany) in accordance with the manufacturer’s instructions. The concentration and purity of RNA were determined by spectrophotometer (Nanodrop 1000, Thermo Scientific). 5 µg of RNA extracted from each tissues sample was reverse-transcribed into cDNA using M-MLV reverse transcriptase kit (Invitrogen) primed with Invitrogen’s protocol. The amount of cDNA corresponding to 20 ng of reverse-transcribed RNA was amplified by RT-qPCR, using specific primers (Tables 2 and 3) RT-qPCR was performed in 25 μL volumes in 96-well microplates using FastStart Essential DNA Green Master Mix (Roche, Germany) The 3-step amplification and signal detection were performed using a LightCycler R^®^ 96 (Roche) with an initial pre-incubation at 95 °C for 10 min followed by 40 cycles of 95 °C for 15 s, 56 °C for the 30 s and 72 °C for 30 s. The β-actin gene was used as a housekeeping gene, to correct for differences in template RNA levels between samples during the experiment. Each RT-qPCR experiment thus included triplicates of 16 test samples, one no-template-control, and a log_10_ dilution series. The mean Ct value was used for subsequent calculations. Expression of the IL-4, IL-6, IL-10, TNF-α, *casp*-3, *casp*-8, *casp*-9, *bax* and *bcl*-2 genes was quantified according to Hangalapura et al. [29] (Table 2). First, the Difference Factor for each sample was calculated by dividing the mean Ct value for the β-actin gene of each individual sample by the mean Ct value for the β-actin gene of all samples. Secondly, the adjusted cytokine mRNA amount per sample was calculated using the following formulae:

$${\text{Difference factor for each Ct sample: [mean Ct value for }}\,{\upbeta} {\text{-actin gene of individual sample/mean Ct value for}}\,{\upbeta}{\text{-actin gene of all samples]}}$$

$${\text{Adjusted cytokine quantity for each Ct sample}}: {\text{[(40-mean cytokine Ct sample)}} \times {\text{cytokine slope]/Difference factor sample}}-{\upbeta}{\text{-actin slope}}$$

**Table 2:** **List of the primers used in RT-qPCR analysis of mRNA expression of selected host genes**

Name	Primer	Primer sequence(5′–3′)	Accession	Conc. (mM)
bcl-2	Forward	GATGACCGAGTACCTGAACC	NM205339	0.2
	Reverse	CAGGAGAAATCGAACAAAGGC		
bax	Forward	TCCTCATCGCCATGCTCAT	XM422067	0.4
	Reverse	CCTTGGTCTGGAAGCAGAAGA		
caspase-8	Forward	TGGCCCTCTTGAACTGAAAG	AY057940	0.4
	Reverse	TCCACTGTCTGCTTCAATACC		
caspase-9	Forward	CGAAGGAGCAAGCACGACAG	AY057940	0.2
	Reverse	CCGCAGCCCTCATCTAGCAT		
caspase-3	Forward	TGGCCCTCTTGAACTGAAAG	AY057940	0.4
	Reverse	TCCACTGTCTGCTTCAATACC		
IL-6	Forward	GCTCGCCGGCTTCGA	AJ250838	0.2
	Reverse	GGTAGGTCTGAAAGGCGAACAG		
IL-4	Forward	AACATGCGTCAGCTCCTGAAT	AJ621735	0.4
	Reverse	TCTGCTAGGAACTTCTCCATTGAA		
IL-10	Forward	CATGCTGCTGGGCCTGAA	J621614	0.4
	Reverse	CGTCTCCTTGATCTGCTTGATG		
TNF-α	Forward	GCCCTTCCTGTAACCAGATG	NM_204267	0.2
	Reverse	ACACGACAGCCAAGTCAACG		
β-actin	Forward	CCGCTCTATGAAGGCTACGC	L08165	0.4
	Reverse	CTCTCGGCTGTGGTGGTGAA

**Table 3:** **Gross pathology and re-isolation rates of*G. anatis*from different organs following experimental infection with a wild-type strain (*G. anatis*12656-12) (4 chickens) or its isogenic*gtxA*deletion mutant (Δ*gtxA*) (16 chickens)**

*G. anatis*	Peritoneum		Ovary		Oviduct	
	Purulent peritonitis	Re-isolation of *G. anatis*	Purulent oophoritis	Re-isolation of *G. anatis*	Salpingitis	Re-isolation of *G. anatis*
Δ*gtxA*	2/16 (13%)	2/16 (13%)	5/16 (31%)	10/16 (63%)	0/16 (0%)	7/16 (44%)
Wild-type	3/4 (75%)	3/4 (75%)	3/4 (75%)	3/4 (75%)	3/4 (75%)	3/4 (75%)

### Statistics

Fisher’s Exact test was used for comparison of differences in the extent of the gross lesions and bacterial re-isolation rates. ANOVA was used to compare antibody-titers. The Kruskal–Wallis test was used for comparisons of histological lesion scores. Differences in gene expression levels were compared by Students *t*-test using GraphPad Prism 7^®^ for Windows (GraphPad, San Diego, USA). *P*-values ≤ 0.05 were considered statistically significant.

## Results

### Pathology induced by *G. anatis* WT strain and its isogenetic Δ*gtxA* mutant

**Figure 1:**
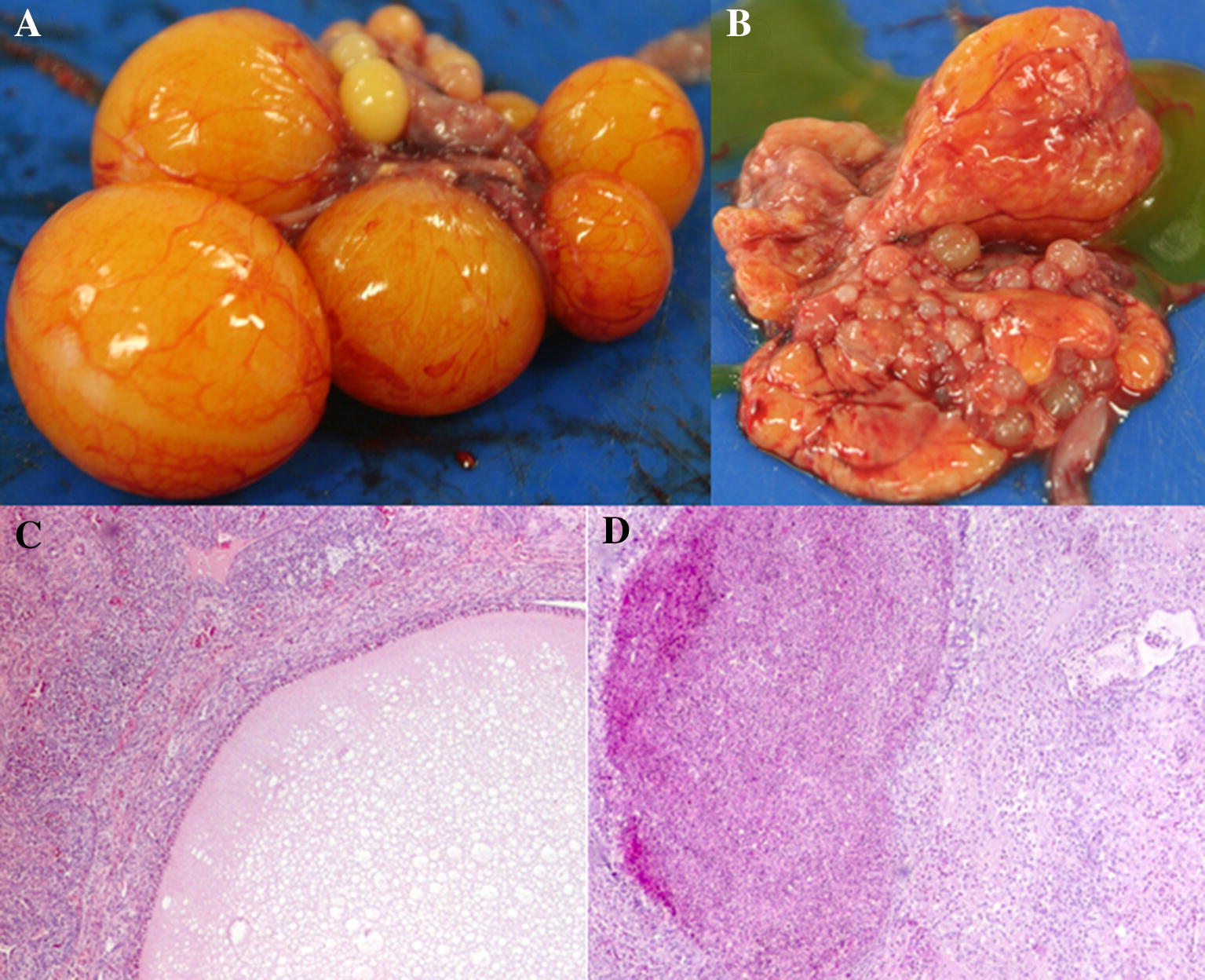
**Gross lesions, histopathology and apoptotic cells found in chickens infected with*G. anatis*Δ*gtxA*mutant or*G. anatis*wild-type (WT) strain.** Ovaries from 29-week old laying hens. Two days post-inoculation with either *G. anatis* Δ*gtxA* or *G. anatis* WT. **A***G. anatis* Δ*gtxA* infection group. Oophoritis with vascular engorgement and slight edema. **B***G. anatis* wild-type. Diffuse purulent oophoritis and folliculitis with ruptures follicles. **C** Hematoxylin and eosin (HE)-straining of the follicle in ovarian tissue from the *G. anatis* Δ*gtxA* infection group. Focal oophoritis with the presence of inflammatory cells in the stroma and vascular engorgement. Infiltrates of few heterophilic granulocytes and mononuclear cells. **D** HE-straining of the ovary of the *G. anatis* WT infection group. Purulent oophoritis with heavy infiltration of inflammatory cells and granuloma formation (black arrows) in the follicle.

**Figure 2:**
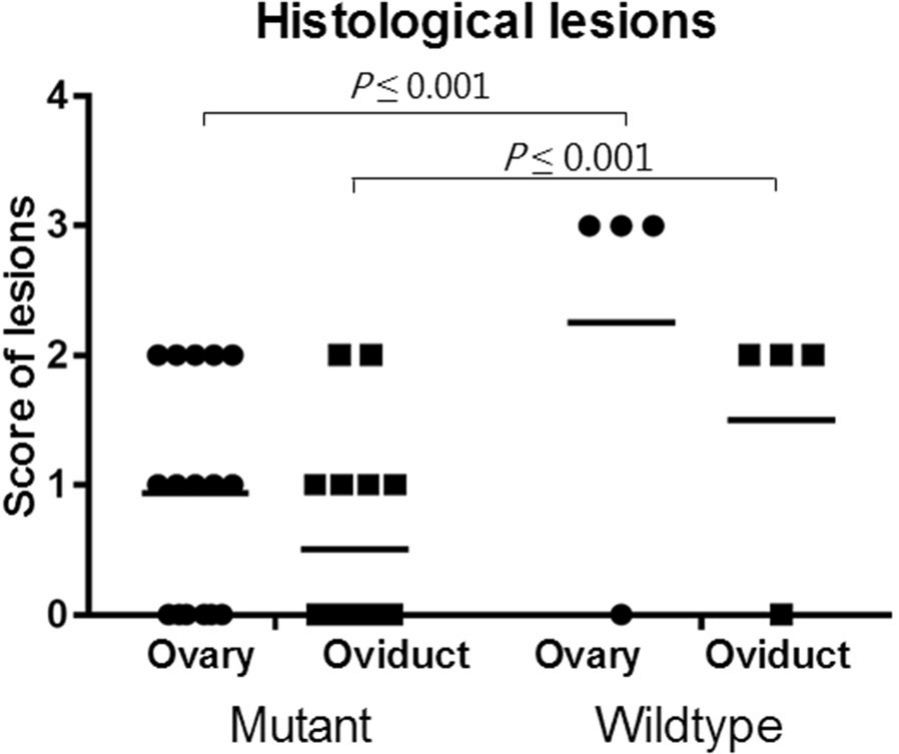
**Score of histological lesions in the ovary and oviduct of chickens infected with*G. anatis*Δ*gtxA*or*G. anatis*wild-type strain.** No clinical signs of infection were observed after inoculation. At the necropsy, 10 out of the 16 birds having received *G. anatis* Δ*gtxA* demonstrated mild lesions, whereas remaining birds did not have any lesions. In birds infected with *G. anatis* WT strain, three of four birds had severe lesions and one was without lesions. No lesions were found in the control bird group. The number of organs with lesions in birds from the Δ*gtxA* group was significantly lower compared to the number of organs with lesions in WT group (*P* = 0.003). No difference was found between a number of lesions when comparing birds examined at 2 or 6 days pi from the Δ*gtxA* group (*P* = 0.2). In the Δ*gtxA* infection group, a mild non-purulent oophoritis characterized by vascular congestion and enlargement of the stromal tissue of the ovary (Figure 1A) was observed in five birds. In three birds, the oophoritis was purulent and in two birds, both examined 6 days pi, focal purulent oophoritis and localized peritonitis was found. Salpingitis was not observed in any of the birds from the Δ*gtxA* mutant infection group. The three out of four birds in the WT strain group had gross lesions, all exhibiting diffuse purulent peritonitis, diffuse purulent oophoritis and salpingitis (Figure 1B). No lesions were found in the spleen or liver of any birds. * indicates a statistically significant difference between groups (*P* ≤ 0.05).

No clinical signs of infection were seen after the bacterial inoculations. At the necropsy, ten out of the 16 birds having received *G. anatis* Δ*gtxA* demonstrated mild macroscopic lesions whereas the remaining chickens had no lesions. Of the chickens infected with *G. anatis* WT, three had severe lesions and one had no lesions. No lesions were found in the birds in the BHI inoculated control group. The number of organs with lesions in birds from the Δ*gtxA* group, was significantly lower compared to the number of organs with lesions in WT group (*P* = 0.003). No difference was found between number of lesions when comparing birds examined at 2 or 6 days pi from the Δ*gtxA* group (*P* = 0.2). The histological lesions in both the Δ*gtxA* and WT infection groups supported the observations noted from the gross lesions (Figure 1). No histological lesions were found in birds without gross lesions. In the Δ*gtxA* group, few focal infiltrations of inflammatory cells, predominantly lymphocytes, were found in the oviduct and in one bird a necrotic focus was found. The ovarian stromal tissue was edematous with blood in the vascular system. Additionally, only light infiltration of inflammatory cells and degeneration of follicles were seen in birds with purulent oophoritis (Figure 1C). In the group infected with the WT strain, multifocal infiltration with inflammatory cells was present in the oviduct and several foci with bacterial colonization were also evident. In the ovary, infiltration with inflammatory cells including lymphocytes and heterophils was identified. In some areas, granuloma formation surrounded by epithelioid cells and necrosis was found (Figure 1D). A significant difference in histological lesion scores of both ovarian (*P* < 0.001) and oviduct tissues (*P* < 0.001) was found when comparing the two groups (Figure 2).

### Re-isolation of *G. anatis*

Resident cloacal *G. anatis* was isolated from 17 of the 24 birds prior to the experimental infections. At the necropsy *G. anatis* was re-isolated in pure culture from all gross lesions in the infected groups. Additionally, *G. anatis* was isolated from the oviduct mucosa of hens of the Δ*gtxA* infected group even in cases where no gross lesions were evident (Table 3). No bacteria were isolated from the internal organs of birds in the control group. To compare the isolates obtained from lesions and from the cloacal mucosa, pairs of isolates were characterized from eleven individual birds. All eleven isolates associated lesions had identical band patterns corresponding to the inoculated strains, whereas all of the cloacal isolates clearly had different PFGE profiles (Additional file 1).

**Figure 3:**
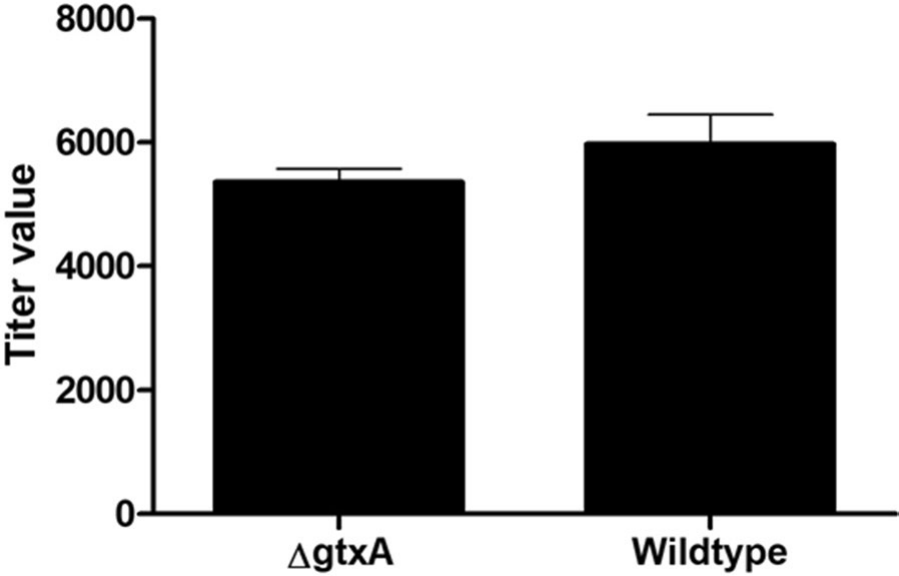
**Titers for GtxA-specific IgG in serum from groups of birds.** Blood drawn at the time of inoculation with either *G. anatis* Δ*gtxA* mutant (*n* = 4) or *G. anatis* wild-type (*n* = 8). No significant difference was found between the groups (*P* = 0.2).

The titer of GtxA specific antibodies was quantified at the time of inoculation and found at background levels in both groups (Figure 3).

### Gene differential expression analysis by real-time quantitative PCR

**Figure 4:**
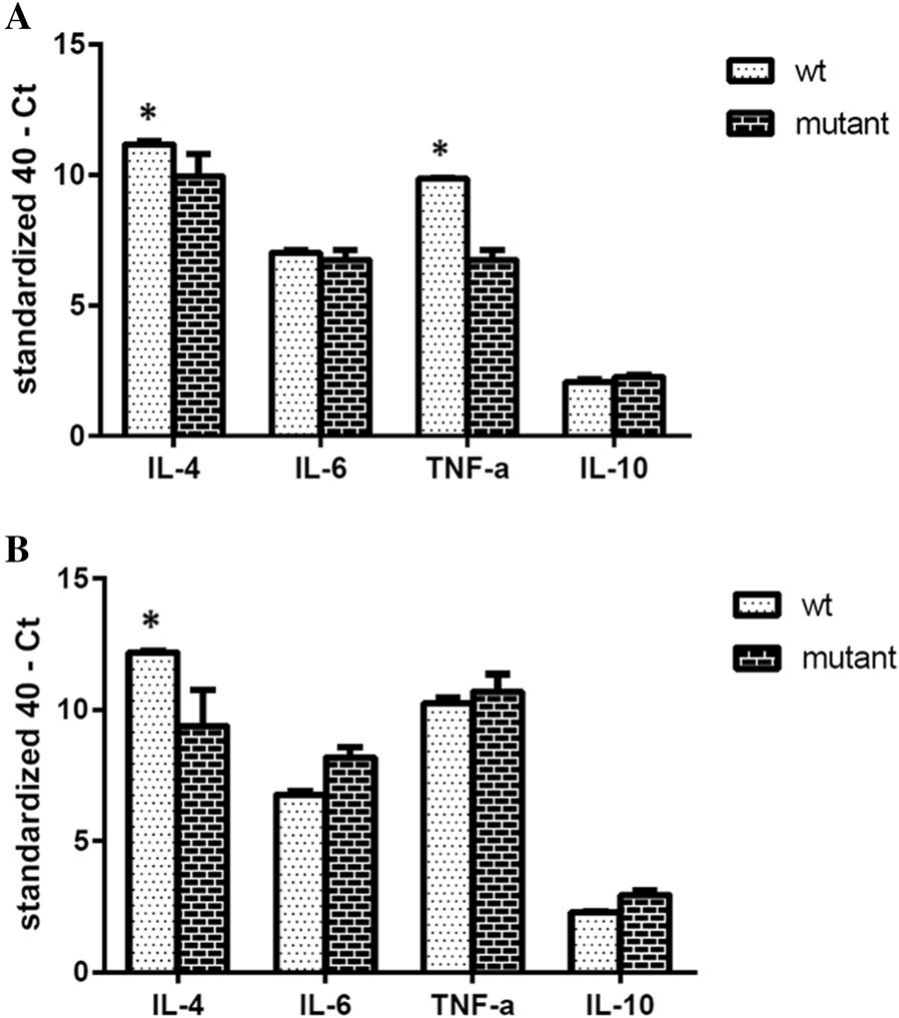
**Changes of the cytokine mRNA expression levels in the ovary.** Cytokine mRNA was isolated from culture positive ovary tissue at 2 days pi (**A**) and 6 days pi (**B**). As values are subtracted from the negative endpoint, higher values represent higher levels of cytokine mRNA levels. Error bars are S.E. for each treatment group (*P < 0.05, **P < 0.01) between WT (white bars) and ΔgtxA mutant groups (black bars).

To assess the ability of GtxA in mounting a host response during the *G. anatis* infection, the expression levels of cytokines IL-4, IL-6 IL-10 and TNF-α were quantified in the ovary and spleen tissues. No differences in the expression of the pro-inflammatory cytokines IL-6 and IL-10 were found in the ovary at 2 days pi between WT strain and Δ*gtxA* (Figure 4A), whereas significantly lower expression of IL-4 and TNF-α, respectively, was found in the Δ*gtxA* group compared to the WT group. At 6 days pi, expression of IL-4 remained significantly lower in the *ΔgtxA* group compared to the WT strain group (Figure 4B), while the ovarian expression of IL-6, TNF-α and IL-10 did not differ between the two infection groups (Figure 4B).

**Figure 5:**
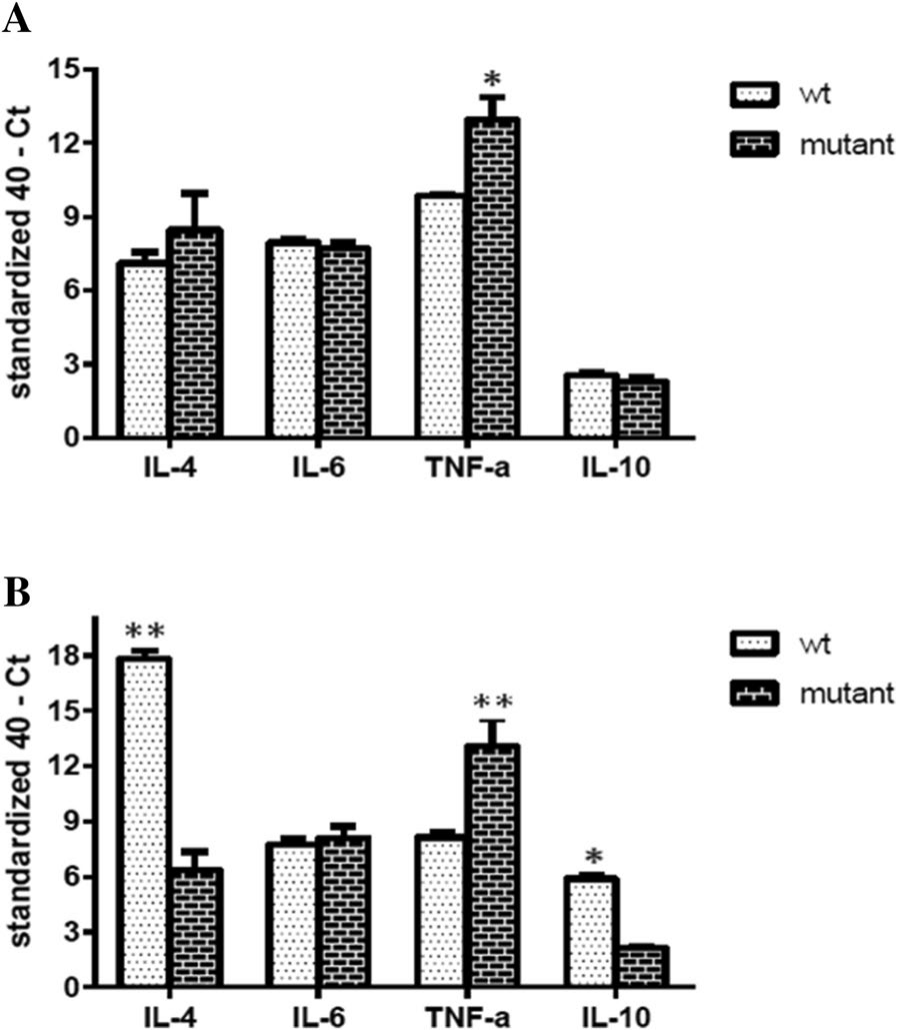
**Changes of the cytokine mRNA expression levels in the spleen.** Cytokine mRNA was isolated from culture positive spleen tissue at 2 days pi (**A**) and 6 days pi. (**B**). Error bars are S.E. for each treatment group (*P < 0.05, **P < 0.01) between WT (white bars) and *ΔgtxA* mutant groups (black bars).

In the spleen, the TNF-α expression was significantly higher in the *ΔgtxA* infected hens compared to the WT group at 2 days pi, while for IL-4, IL-6 and IL-10 no statistically significant difference was found between the two groups (Figure 5A). At 6 days pi, the expression of IL-4 and IL-10 in the spleen tissue was significantly lower in the *ΔgtxA* group compared to the WT group, while TNF-α expression was significantly higher in the Δ*gtxA* group (Figure 5B). In all tissue samples from WT group and the Δ*gtxA* group the level of IFN-γ was not detectable (data not shown).

### Apoptosis-related gene expression in the ovary

**Figure 6:**
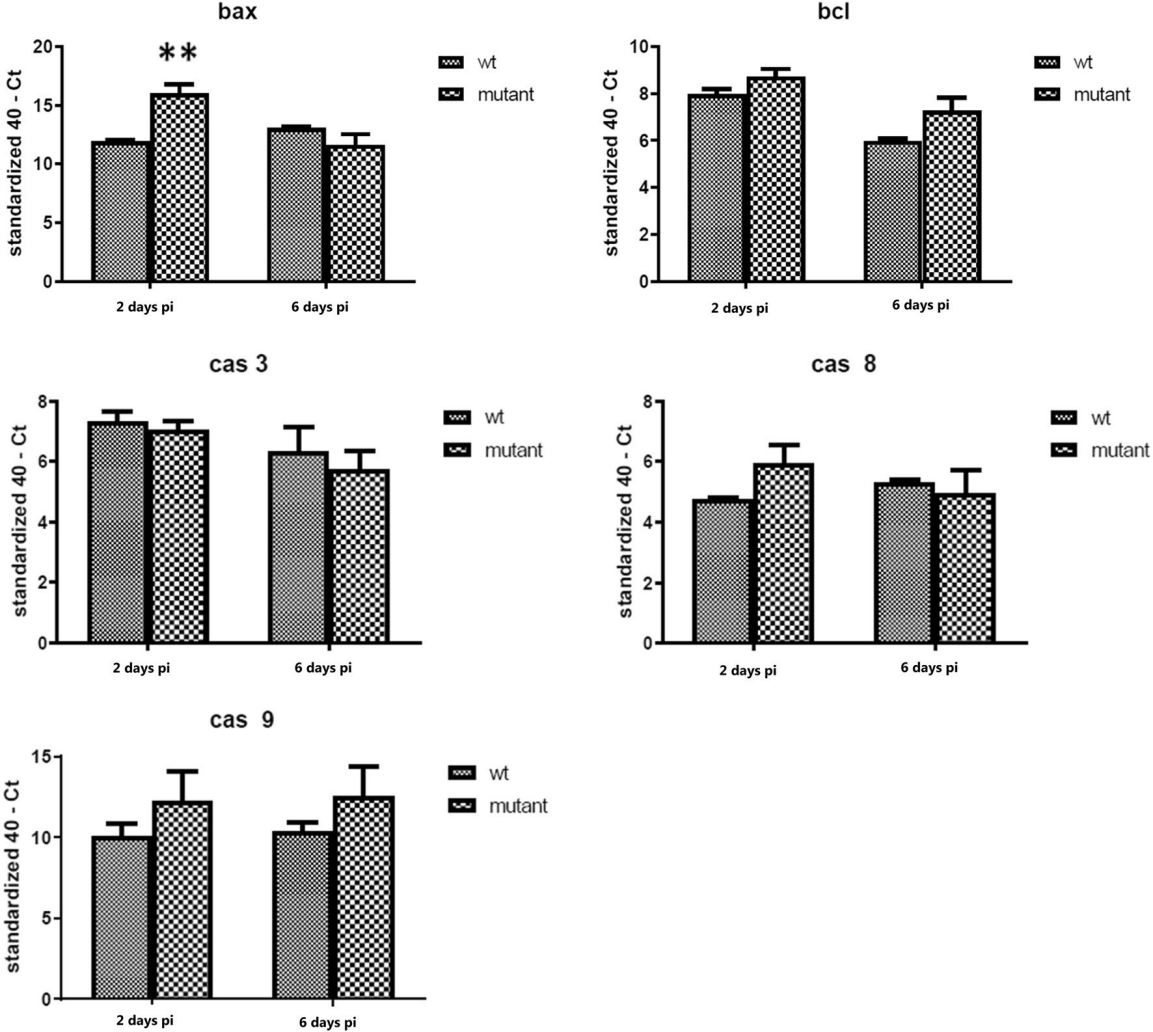
**The relative expression levels of apoptosis genes in ovary tissue at 2** **days and 6** **days post-infection of*gtxA*mutant compared with WT strain.** Error bars are S.E. for each treatment group The figure shows the gene expression profile of apoptosis-related (*Bcl*-2), *caspase*-8, *caspase*-3, *caspase*-9 differed significantly (*P < 0.05, **P < 0.01) between the WT strain and Δ*gtxA* mutant groups, respectively.

The expression of selected apoptosis genes was evaluated in the ovarian tissue where the expression of *bax* was significantly increased in the Δ*gtxA* infection group compared to the WT group at 2 days pi (Figure 6). At 6 days pi, the expression the apoptosis genes was not different between the Δ*gtxA* and WT group, respectively (Figure 6).

### Apoptosis-related gene expression in the spleen

**Figure 7:**
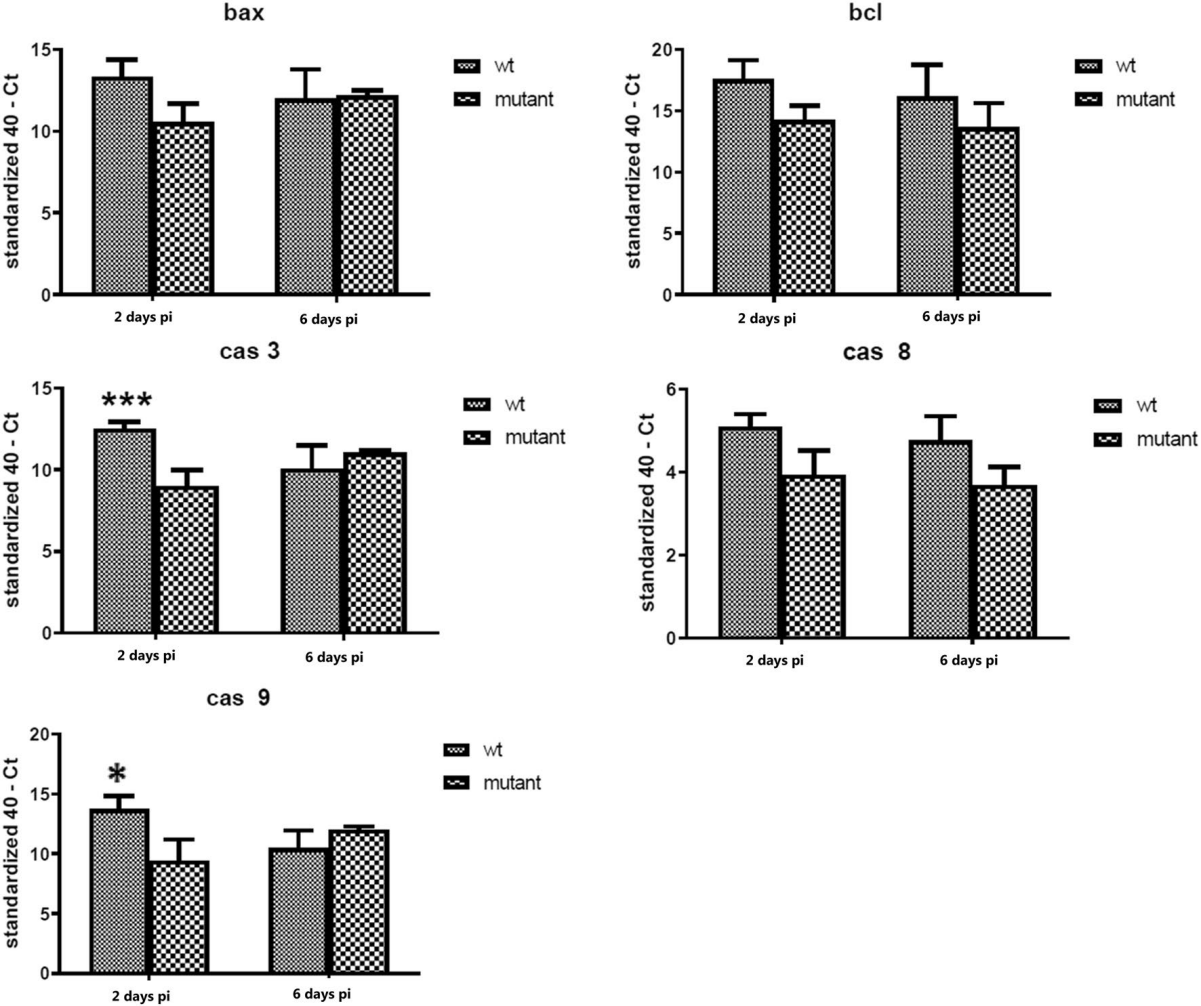
**The relative expression changes of apoptosis genes in the spleen at 2** **days and 6** **days pi with the*gtxA*mutant compared with WT strain, respectively.** Error bars are S.E. for each treatment group The gene expression profiles of apoptosis-related mRNA of B cell lymphoma-2 (*Bcl*-2), *caspase*-8, *caspase*-3, *caspase*-9 differed significantly (*P < 0.05, **P < 0.01, ***P < 0.001) between WT and Δ*gtxA* mutant groups, respectively.

The expression of apoptosis genes in the spleen tissue was assessed at 2 days and 6 days pi (Figure 7). The expression of *bax*, *bcl*-*2*, *casp*-*3*, *casp*-*8* and *casp*-*9* were all numerically lower at 2 days pi in the *gtxA* mutant group compared with WT group, yet this was only significant for *casp*-*3* and *casp*-*9* (Figure 7). At 6 days pi, no statistically significant difference in the expression was observed between the Δ*gtxA* infected group and the WT group (Figure 7).

## Discussion

The results of the present study showed that the virulence of *G. anatis* was severely decreased in vivo when expression of the *gtxA* gene was impaired. Loss of virulence was indicated by a significantly lower lesion score in birds infected with the Δ*gtxA* strain compared to those infected with the GtxA-producing strain (Figures 1 and 2). The lesions induced by the *G. anatis* WT strain corresponded closely with observations made from previous in vivo studies [2, 14, 27], and thus justified the limited number of chickens used in the WT group. One WT infected hen had no apparent lesions and the inoculum had seemingly been deposited and trapped in a lump of abdominal fat. The observed differences in severity of lesions are in accordance with in vivo studies investigating strains of *Actinobacillus pleuropneumoniae* lacking the ability to produce different RTX-toxins either due to inactivation or deletion of the toxin genes, which all showed a sharp decline in virulence including lower mortality, morbidity and diminished lesions in pigs [31–33]. Experimental infection with a *lktA* (a leukotoxin gene) deleted mutant of *Mannheimia haemolytica* also led to a decrease in clinical signs and lung lesions in calves when compared to the isogenic WT strain [34]. Hence our findings support previous observations of RTX-toxins produced by other members of *Pasteurellaceae* and stress the profound role of GtxA in the pathogenesis of *G. anatis.*

Most RTX-toxins seem to enforce a strong leukotoxic effect and thereby debilitating the host immune response, which seems particularly aimed at specific host cells and thereby a likely cause of the host-specific pathogenicity commonly observed among *Pasteurellaceae* bacteria [9, 35]. In the present study, the inflammatory response promoted by the Δ*gtxA* mutant was significantly reduced whereas the WT induced severe purulent oophoritis with a high number of heterophilic granulocytes present and granuloma formation, indicating that GtxA plays an important role at activating heterophils during the immediate inflammatory response against *G. anatis*. Previous reports have indicated a similar role of the heterophils during other bacterial infections [36–38].

The highly decreased cellular infiltration and inflammatory reaction found in the tissues of the birds infected with the Δ*gtxA* mutant (Figures 1A and C) indicated less tissue destruction in the absence of GtxA, subsequently leading to a weaker host response. Virulence factors, including RTX-toxins, of other species of *Pasteurellaceae* have been shown to boost a strong pro-inflammatory response in vitro [36] and in vivo [2] and the lack of GtxA expression might similarly, lead to a diminished inflammatory response. This is, at least partly, supported by the re-isolation of the Δ*gtxA* mutant from the oviduct without a concurrent inflammatory response. However, it also suggests that *G. anatis* unability to produce GtxA withstands the capacity to effectively colonize the chicken oviduct and thereby points at other bacterial factors e.g. the F17-like fimbria that may take part in this process [12, 40, 41].

Due to the widespread occurrence of *G. anatis* among laying hens, it was not surprising that *G. anatis* could be isolated in the cloacae of most of the chickens entering the experiment. However, the strains isolated from the lesions associated with the experimental infections all showed to be identical to the strains used for the inoculation and different from the *G. anatis* isolates from the cloaca (Additional file 1). The factors permitting a commensal lifestyle of *G. anatis* are not well understood, but the present results indicate that the bird’s immune system generally have not been stimulated to initiate an adaptive response in healthy carriers as the GtxA specific titers were at or below the background level, as previously reported [14, 27]. Overall, the *G. anatis* Δ*gtxA* mutant was strongly attenuated upon experimental infection in its natural host suggesting that GtxA contributes significantly to the severity of infections in chickens.

Previous studies have clearly indicated GtxA to be a virulence factor while having the ability to induce protective immunity against *G. anatis* [27]. However, the specific host immune responses, here represented by cytokine and apoptosis gene expression profiles, in poultry have not previously been reported. In the current study we investigated pro-inflammatory cytokines involved in systemic inflammation and stimulation of the acute phase reaction [29, 36, 41]. We observed a high level of IL-4 and TNF-α mRNA expression in the ovary at 2 days and 6 days pi in the WT group, but a relatively low level in the spleen tissue at 2 days pi (Figures 4 and 5). The results are consistent with those reported by Zhang et al. [18], who revealed that *G. anatis* could induce marked inflammatory responses in primary chicken oviduct epithelial cells. The results indicate that GtxA may be involved in the stimulation of an IL-4 and TNF-α and promotes Th2-like response particularly in the ovary tissue, which previously has been suggested a main target for *G. anatis* [39].

As a pro-inflammatory cytokine, IL-6 is involved in the recruitment of immune cells, including lymphocytes and circulating monocytes, to the site of infection [17]. In our study, there were no significant differences in the IL-6 mRNA expression when comparing the WT and the Δ*gtxA* mutant in the ovary or the spleen tissues, respectively (Figures 4 and 5). It may be that in chickens, GtxA modulates the adaptive rather than innate immune response.

The regulation of the Th1-Th2 cytokine ratio plays an important role in balancing the host immune response [42, 43]. Our results indicated that GtxA expression primarily initiated a Th2-like response, as indicated by the increased IL-4 expression at 2 days and 6 days in the ovary tissue and similar but slightly delayed response (only at day 6) in the spleen tissue. On the contrary, in the Δ*gtxA* infected group, TNF-α was highly expressed, particularly in the spleen tissue at 2 days and 6 days whereas the level of IL-4 was low. These results indicate that the Δ*gtxA* mutant is able to inhibit or simply unable to induce secretion of IL-4. IL-4 is produced by Th2 cells that take part in the regulation of humoral immunity in poultry [44]. The cytokine expression levels are in good accordance with our pathological findings, showing a highly decreased cellular infiltration and inflammatory reaction in the tissues of birds infected with Δ*gtxA* (Figures 1A and 1C). The results also indicated that the Δ*gtxA* mutant induced less tissue and cellular destruction, subsequently leading to a weaker host response possibly through partial impairment of the Th2-like pathway. IL-10 is an anti-inflammatory cytokine [45, 46], negatively affecting the expression of Th1 cytokines [41]. We found that tissues from the WT infected group also had increased expression of IL-4 and partly IL-10 but not IFN-γ (which was below the detection level in all samples examined), supporting the Th2 bias suggested.

We hypothesized that GtxA was involved in regulating the apoptotic response in the host. Two major forms of apoptosis are commonly recognized, one involving activation of *casp*-8 (external induction) through the “death receptor” in the plasma membrane and the other involving disruption of the host cell homeostasis (intrinsic induction) by *casp*-9 regulation [47]. Both initiator caspases can recruit the executioner casp-3, which degrades cellular targets during apoptosis [47]. RTX toxins, such as *Staphylococcus aureus* toxin (alpha) and *Actinobacillus* leukotoxin, at low concentrations, have been suggested to cause the formation of small pores in the host cell plasma membrane allowing influx of Ca^2+^, which activates the intrinsic apoptotic pathways [48–50]. In our study, *casp*-3, -8, -9, *bax* and *bcl*-*2* expression was significantly lower in the spleen tissue at 2 days pi in the chickens challenged with the Δ*gtxA* mutant compared to the WT (Figure 7), while the level of TNF-α was elevated (Figure 5A). This may reflect a dose–response relation where some but only a limited number of WT bacterial cells reached the spleen as opposed to the Δ*gtxA* mutant where very few if any bacterial cells made it to the spleen. On the contrary, in the ovary tissue, Δ*gtxA* induced higher levels of some of the pro-apoptosis genes (*casp*-*8*,-*9* and *bax*) at 2 days, whereas apoptosis suppression, induced by increased *bcl*-2 expression, seemed apparent at 6 days (Figure 6B). The results are consistent with the fact that we observed only mild or no macro- and microscopic lesions in the ovary of the Δ*gtxA* infected chickens despite presence of bacteria in 10 out of 16 birds (Table 3). Based on these observations the Δ*gtxA* mutant seem able of inducing an apoptotic host response, which may allow prolonged survival in the host. At a high exposure of ovary tissue to the WT and GtxA a clear inflammatory response was observed. GtxA presumably forms large pores and rapid cytolysis of phagocytic cells [10], allowing the bacteria to escape the host defenses, which may prolong bacterial survival and increase the severity of the disease. The possible RTX toxin-induced pro-inflammatory death pathway, is a mechanism employed by *Salmonella enterica*, serovar Typhimurium and some *Shigella* species by initiating *casp*-1 expression through the lymphocyte function-associated antigen-1 (LFA-1) pathway [50–52]. Further research is however needed on how TNF-α is induced by non-GtxA expressing bacterial cell and how that may be involved in the regulation of apoptosis in cells exposed to *G. anatis*. Host cells may exploit the apoptosis process as a primitive defence mechanism against bacterial infections. On the other hand, several bacteria seem to have evolved mechanisms to prevent or delay apoptosis of host cells to permit successful bacterial replication [45]. GtxA may thus be involved in apoptosis suppression to facilitate *G. anatis* multiplication in the ovary at an early stage, which is considered a preferred site of this bacterium [39].

This study is the first investigation to assign a specific role of GtxA in the outcome of an infection of the natural host. It was documented that GtxA is important for the severity of lesions in an biologically relevant in vivo experiment and that GtxA contributes to stimulating both the innate and parts of the adaptive cellular immune system through primarily a Th2 response. Finally, our results indicated GtxA to be involved in the induction of apoptosis-related genes in spleen tissue.

## Supplementary information

**Additional file 1. Pulsed-Field Gel Electrophoresis typing of resident*G. anatis*isolates and strains used in the inoculum.** The 12656-12 wild-type (WT) strain and *gtxA* mutant (M) strains. Cloacal isolates (C) and isolates obtained post-inoculation from organs with lesions (L). WT, M and L strains have identical genotypes whereas the cloacal isolates belong to a genetically different group.

## Data Availability

The datasets generated and analysed during the current study are available from the corresponding author on reasonable request.
