# Supplementary figures and images for: GtxA is a virulence factor that promotes a Th2-like response during Gallibacterium anatis infection in laying hens
Source: Vet Res. 2020 Mar 11;51:40. doi: 10.1186/s13567-020-00764-2 (PMC7065373; doi:10.1186/s13567-020-00764-2)

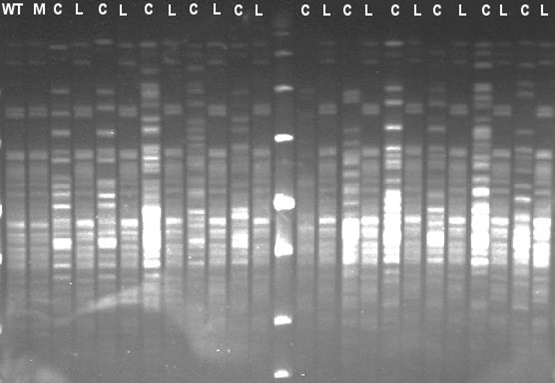

Supplement: Supplementary file 1 — Additional file 1. Pulsed-Field Gel Electrophoresis typing of residentG. anatisisolates and strains used in the inoculum. The 12656-12 wild-type (WT) strain and gtxA mutant (M) strains. Cloacal isolates (C) and isolates obtained post-inoculation from organs with lesions (L). WT, M and L strains have identical genotypes whereas the cloacal isolates belong to a genetically different group. [file 13567_2020_764_MOESM1_ESM.tif]
